# The *thiG* Gene Is Required for Full Virulence of *Xanthomonas oryzae* pv. *oryzae* by Preventing Cell Aggregation

**DOI:** 10.1371/journal.pone.0134237

**Published:** 2015-07-29

**Authors:** Xiaoyue Yu, Xiaoyu Liang, Kexue Liu, Wenxia Dong, Jianxin Wang, Ming-guo Zhou

**Affiliations:** College of Plant Protection, Nanjing Agricultural University, Nanjing, Jiangsu Province, China; Indian Institute of Science, INDIA

## Abstract

Bacterial blight of rice is an important serious bacterial diseases of rice in many rice-growing regions, caused by *Xanthomonas oryzae* pv. *oryzae* (*Xoo*). The *thiG* gene from *Xoo* strain ZJ173, which is involved with thiazole moiety production in the thiamine biosynthesis pathway, is highly conserved among the members of *Xanthomonas*. The *thiG* deletion mutant displayed impaired virulence and growth in thiamine-free medium but maintained its normal growth rate in the rice tissues, indicating that the *thiG* gene is involved in *Xoo* virulence. Compared to the wild type strain, the formation of cell-cell aggregates was affected in *thiG* deletion mutants. Although biofilm formation was promoted, motility and migration in rice leaves were repressed in the *thiG* mutants, and therefore limited the expansion of pathogen infection in rice. Quorum sensing and extracellular substance are two key factors that contribute to the formation of cell-cell aggregates. Our study found that in the *thiG* mutant the expression of two genes, *rpfC* and *rpfG*, which form a two-component regulatory signal system involved in the regulation of biofilm formation by a second messenger cyclic di-GMP is down-regulated. In addition, our study showed that xanthan production was not affected but the expression of some genes associated with xanthan biosynthesis, like *gumD*, *gumE*, *gumH* and *gumM*, were up-regulated in *thiG* mutants. Taken together, these findings are the first to demonstrate the role of the thiazole biosynthsis gene, *thiG*, in virulence and the formation of aggregates in *Xanthomonas oryzae* pv. *oryzae*.

## Introduction

Thiamine, or vitamin B1, is present in animals, plants and microorganisms as free thiamine or as the phosphorylated form thiamine pyrophosphate (TPP). The thiamine pathway is well understood in prokaryotes [1], and several enzymes in thiamine biosynthesis pathway in prokaryotes have been identified. Thiamine is formed by the coupling of two moieties, 5-(2-hydroxyethyl)-4-methylthiazole phosphate (HET-P) and 4-amino-5-hydroxymethyl-2-methylpyrimidine diphosphate (HMP-PP). The hydroxymethyl pyrimidine synthase (ThiC, EC: 4.1.99.17) catalyzes aminoimidazole ribotide (AIR) to form hydroxymethl pyrimidine phosphate (HMP-P), and then HMP-P is phosphorylated to HMP-PP by hydroxymethyl pyrimidine (phosphate) kinase (ThiD, EC: 2.7.4.7). Another moiety HET-P is formed from cysteine, tyrosine, and 1-deoxy-D-xylulose phosphate by the thiazole biosynthesis protein (ThiG, EC: 2.8.1.10). These thiazole and pyrimidine substrates then form thiamine monophosphate by thiamine-phosphate pyrophosphorylase (ThiE, EC: 2.5.1.3), with thiamine monophosphate ultimately being phosphorylated into TPP by thiamine monophosphate kinase (ThiL, EC: 2.7.4.16).

The genus *Xanthomonas* comprises a group of important and ubiquitous Gram-negative plant-pathogenic bacteria [2]. *Xoo* is the pathogen of bacterial blight of rice, which is the most serious bacterial disease of rice in many rice-growing regions [3]. In recent years, many studies on *Xoo* focus on investigating new genes or pathways associated with pathogenicity/virulence [4], and bacterium-plant interactions, but limited attention was focused on the thiamine biosynthesis related genes. By conducting extensive BLAST searches querying the available genome data base of *Xoo* strain PXO99A (GenBank accession number NC_010717) using the respective homologous enzymes from other organisms, we identified the open reading frames (ORFs) of ThiC (*thiC*, YP_001915417), ThiD (*thiD*, YP_001912869), ThiE (*thiE*, YP_001915211), ThiG (*thiG*, YP_001914750), and ThiL (*thiL*, YP_001912199) (unpublished data), which suggests there may be a *de novo* thiamine biosynthesis pathway in *Xoo*.

Recently, several studies reported additional functions for the genes involved in thiamine biosynthesis. The knockout of *thiD* in *Listeria monocytogenes* reduced the replication of the bacterium in epithelial cells and thereby inhibited its infection of host cells [5]. By screening a Tn-5 tagged library of *Xanthomonas oryzae* pv. *oryzicola*, *thiE* was listed as a virulence factor [6]. The protein encoded by *thiG* acts as an essential enzyme in thiazole moiety synthesis in the thiamine biosynthesis pathway, and other studies revealed additional cellular functions of ThiG, such as tolerance to oxidative and heat stress in *Fusarium oxysporum* [7] and maintenance of mitochondrial genome stability in *Saccharomyces cerevisiae* [8], in addition to being required for full pathogenicity in *Verticillium dahlia* [9]. In this paper, we are the first to define the *thiG* gene as a virulence factor in *Xoo*, and demonstrate that the loss of virulence is caused by the formation of cell-cell aggregates, which is associated with quorum sensing and xanthan synthesis in *Xoo*.

## Materials and Methods

### Plant, bacterial and their culture conditions

Rice cultivar IR24 was grown in the greenhouse at 20–25°C. Plasmids and bacterial in this study are showed in Table 1. *Xoo* were grown at 28°C in NA medium (yeast extract, 1 g/L; tryptone, 5 g/L; beef extract, 3 g/L; sucrose, 10 g/L; agar,15 g/L) and NB medium (NA without agar). *Escherichia coli* strains were cultured in LB medium at 37°C [10]. The antibiotic kanamycin was added in LB medium at the final concentration of 25 μg/ml.

**Table 1:** Strains and plasmids used in this study.

Strais/plasmid	Characteristics	Reference or source
***Escherichia coli***		
**DH5α**	F^-^,Ф80d*lacZ* ΔM15Δ (*lacZYA-argF*)U169 *endA1 deoR recA1 hsdR17*(r_K_^-^m_K_^+^) *phoA supE44*λ^-^ *thi-l gyrA96 relA1*	Lab collection
***Xanthomonas oryzae* pv. *oryzae***		
**ZJ173**	Laboratory wild type strain	Lab collection
**Δ*thiG***	A *thiG* knock-out mutant of strain ZJ173	This study
**CΔ*thiG***	Δthi*G* harboring pUFR–*thiG*, Km^R^	This study
**Δ*gumE***	A *gumE* knock-out mutant of strain ZJ173	Lab collection
**Δ*gumM***	A *gumM* knock-out mutant of strain ZJ173	Lab collection
**Δ*gumH***	A *gumH* knock-out mutant of strain ZJ173	Lab collection
**plasmid**		
**pKMS1**	Suicide vector derivative from pK18mobGII, sacB^+^, Km^r^	[41]
**pK-*thiG***	A 1034 bp fusion cloned in pKMS1 for a 1236 bp deletion of *thiG*, Km^r^	This study
**pUFR034**	*IncW*, Nm^R^, Km^R^, *Mob* ^+^, *Mob(p)*, *lacZ* alpha, PK2 replicon, cosmid	[42]
**pUFR-*thiG***	A 1300bp fusion cloned in pUFR034 including *thiG*	This study

Km^r^ = kanamycin resistance

### Sequence analysis

A BLASTP search of the NCBI database (http://blast.ncbi.nlm.nih.gov/Blast.cgi) was conducted using the amino acid sequence of ZJ173 *thiG* as a query. Multiple alignments of protein sequences were carried out using ClustalX2, and the phylogenetic tree was constructed using MEGA 4.0 [11].

### Construction of *thiG* deletion mutant and complementation of the *thiG* mutant

The *thiG* deletion mutant was generated by non-marker homologous recombination [12], and pKMS1 was using as a suicide vector. The upstream and downstream fragments of *thiG*, were generated by PCR using the genomic DNA of the wild type strain ZJ173 as the template and the primers listed in S1 Table. The upstream region was digested with *BamH*I and *Kpn*I, and the downstream region was digested with *Kpn*I and *Sal*I. The two fragments were ligated and cloned in the vector pKMS1 previously digested with *BamH*I and *Sal*I, resulting in pK-Δ*thiG* (Table 1). This vector was transformed into ZJ173 competent cells using electroporation, and then the transformants were selected on NA plates with kanamycin but no sucrose. The positive colonies were then transferred to 30ml NB medium without sucrose. After culturing 9 h at 28°C, 200μl of suspension were plated on NA plates with 100g/L sucrose. The positive colonies that grew within 3–4 day were transferred to NA and NA with kanamycin plates, respectively. The colonies sensitive to kanamycin were potential mutants that were subsequently confirmed by PCR amplification and Southern hybridization (Roche, USA) with the primer pair in S1 Table. The confirmed mutant, Δ*thiG* (Table 1), was used for further study.

The complemented mutant was constructed as described previously [12], amplified products of *thiG* and its predicted promoter were generated from the genome DNA of ZJ173 with the primers listed in S1 Table. These fragments were digested with *Kpn*I and *BamH*I and then cloned in pUFR034 previously digested with the same enzymes, resulting in the complemented plasmid pUFR–*thiG* (Table 1). The vector pUFR–*thiG* was transformed into *ΔthiG* competent cells using electroporation and then the complemented strains of *ΔthiG* were selected on NA plates with kanamycin.

### Pathogenicity test

The susceptible rice cultivar IR24 is used for *Xoo* bacterial strain inoculation. The rice leaves were clipped with sterile scissors dipped in the bacterial suspension (OD_600_ = 1.0) [13].The leaves were clipped with sterile water as control. Disease symptoms were measured on 14th day after inoculation. Each treatment has replicate plants and this experiment was performed three times.

### Determination of bacterial growth in the limited nutrient medium, rich nutrient medium and in rice leaf tissues

The growth of the *Xoo* strains were measured both *in vivo* and *in vitro* as described previous [14]. Cells were collected when OD_600nm_ value reached 0.6 by centrifugation for 1 min at 10000 rpm and resuspended in same volume of sterile water, 3 ml of the cell suspension was added to 100 ml of the limited nutrient medium MMX ((NH_4_)_2_SO_4_, 2 g/L; MgSO_4_·7H_2_O, 0.2 g/L; KH_2_PO_4_, 6 g/L; K_2_HPO_4_, 4 g/L; trisodium citrate, 1 g/L; glucose, 5 g/L pH 7.0) and rich nutrient medium NB. The cultures were grown at 28°C and the OD_600nm_ value was measured every 12 h until bacterial growth reached the stationary phase. Each strain has three replicate flasks and this experiment was repeated three times.

The growth rate of *Xoo* strain in rice tissues used the susceptible rice cultivar IR24 as the host plant. The bacterial cell was collected by centrifugation and resuspended in equal volume sterile water. Each plant has five leaves for inoculation with *Xoo* strains. The infiltrated leaves were cut into 6 mm sections around the inoculation spots at different time point after inoculation. After sterilized with 70% ethanol, the leaf sections were grind in 1ml sterile water and diluted in serial concentration, and plated onto NA plates. Each plate was cultured in 28°C incubator and counted the number of colonies after 2–3 days. Each sample was plated onto three plates and this experiment was repeated three times.

### Extracellular polysaccharide (EPS) production, biofilm formation, bacterial migration and motility assays

*Xoo* strains were grown in NB medium for 72h with shaking at 175 rpm at 28°C. EPS in the supernatant was quantified by the colorimetric method used for determination of pentoses and hexoses [15], and was calculated with a standard curve constructed with known amounts of xanthan. Each strain has 3 replications and this experiment was repeated three times.

The method of biofilm formation assay was described by O’Toole and Kolter [16]. Briefly, *Xoo* strains were grown in NB liquid at 28°C with shaking at 175 rpm for about 24h. Cells were harvested when OD_600nm_ reached 0.6 by centrifugation for 1 min at 10000 rpm and resuspended in same volume of sterile water, and then 50 μl of the suspension was added to 2ml of NB in glass tubes. The cultures were kept at 28°C without shaking for 5 days. The cultures were removed from tubes and added an equal volume of 1% (w/v) crystal violet staining for 15 min. After removing the unbound dye with H_2_O, 90% ethanol was utilized for dissolving the dye bounded on the glass and quantified at 590 nm by spectrophotometry.

For motility assays, 2μl suspension grown at the early logarithmic phase were inoculated on the NB plates with 0.3% (w/v) agar and cultured in 28°C incubator [17]. The motility zone was measured 48 h after incubation. Each strain has three replications and the experiment was performed three times.

The bacterial migration assays was described by Chatterjee. S and Sonti R.V. [18]. The leaves from the susceptible rice cultivar IR24 were dipped in 200ml of sterile water with 0.1% glucose to keep the detached leaves in a fresh condition and maintained for 24h at room temperature prior to inoculation with *Xoo* strains by leaf clip method as described above. Infected leaves were sterilized by 2% sodium hypochlorite for 2 min and were washed twice in sterile water at 3d after inoculation. The site of inoculation was identified to be the top of the leaf, and 1cm pieces of the sterilized leaves were cut from the bottom to the top. Then these pieces were incubated on NA medium plates and cultured at 28⁰C for 3–4 days. The distance of bacterial migration was estimated by the colonies formed on NA plates. All the bacteria strain were inoculated in 15 leaves and the experiments performed three times.

### Real-time Reverse Transcription PCR

The relative expression level of tested genes was measured by real-time PCR with the primers shown in S1 Table. The *Xoo* strains were inoculated in 30 ml NB; when the OD_600nm_ value of 0.6 was attained (after 24–36h) and bacterial cells were collected from 1 ml of the suspension. Total RNA of *Xoo* strain was extracted using the RNA prep pure Cell/Bacteria Kit (TIANGEN, China) according to the manufacturer’s protocol. Then, reverse transcription was performed with 1 μg of each RNA sample using cDNA Synthesis kit (TaKaRa, China). Real-time quantitative RT-PCR was performed with iTaq Universal SYBR Green Supermix (Biorad, USA) on the Applied Biosystems 7500 real-time PCR System and with the following thermal cycle conditions: denaturing at 95°C for 30 s followed by 40 cycles of 95°C for 5 s and 60°C for 34 s. The *gyrB* gene was used as the endogenous control. All the experiments were repeated three times.

## Results and Discussion

### The *thiG* gene is highly conserved within the *Xanthomonas* genus

**Fig 1:**
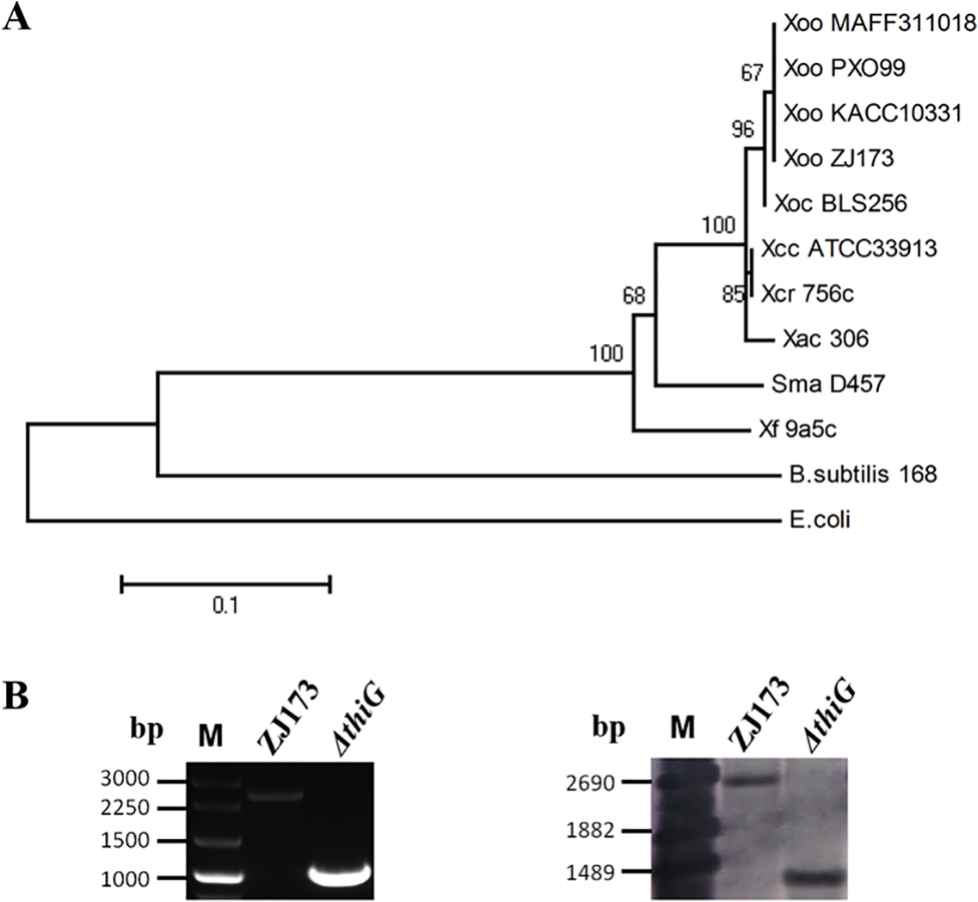
Phylogenetic analysis of *thiG*, ∆*thiG* construction, and its molecular confirmation. (A) Phylogenetic analysis of *thiG* within the family Xanthomonadaceae and model bacterial strains Abbreviations: Xoo, *Xanthomonas oryzae* pv. *oryzae*; Xoc, *Xanthomonas oryzae* pv. *oryzicola;* Xac, *Xanthomonas axonopodis* pv. *citri*; Xcc, *Xanthomonas*. *campestris* pv. *campestris*; Xcr, *Xanthomonas*. *campestris* pv. *Raphani*; Sma, *Stenotrophomonas maltophilia*; Xf, *Xylella fastidiosa;* E. coli, *Escherichia coli;* B. subtilis, *Bacillus subtilis*. The scale bar (0.1) means 10% sequence divergence. Bootstrap values are a value for the significance of the branches. (B) PCR confirmation and Southern blot analysis of *thiG* mutant. The deletion of the ORF for *thiG* resulted in the amplification of a 1000 bp fragment of the deletion mutants. M: mark. The primers used to amplify the probe for the Southern blot are listed in Table 1. DNA fragments of approximately 1400 bp were detected in the *thiG* deletion mutant, whereas the corresponding fragments detected in the wide-type strain ZJ173 were 2500bp.

A phylogenetic analysis was performed by MEGA 4.0 using the neighbor-joining method [11] (Fig 1A). The amino acid sequence of the ThiG protein in the *Xoo* strain ZJ173 is almost identical (99% identity) to the reported ThiG protein from the genomes of the *Xoo* strains, PXO99A, KACC10331, and MAFF311018, indicating that the study of *thiG* gene in *Xoo* strain ZJ173 is representative of all the *thiG* genes in *Xoo*. The thiazole biosynthesis protein ThiG was highly conserved in the Xanthomonadaceae (87–99% identity), including *Xanthomonas*, *Xylella*, and *Stenotrophomonas*. The ThiG protein shares a lower degree of identity with homologues from *E*. *coli* (51%) and *B*. *subtilis* (55%) compared with those in Xanthomonadaceae. However, we assumed that the gene *thiG* in *Xoo* may have a similar function in the thiamine biosynthesis pathway as that reported for *E*. *coli* or *B*. subtilis [19]. To facilitate the functional study of this gene in *Xoo*, we constructed the nonpolar *thiG* deletion mutant, *ΔthiG* (Table 1), by homologous suicide plasmid integration. Putative deletion colonies were examined by PCR and Southern blotting (Fig 1B). The complemented strain, *CΔthiG*, was also constructed by introducing into the mutants the recombinant plasmid pUFR–*thiG*, which carried the entire ORF and the promoters of *thiG* (Table 1).

### The *thiG* gene is required for the full virulence of *Xoo* in rice and suppresses growth in minimal medium but not in rice

**Fig 2:**
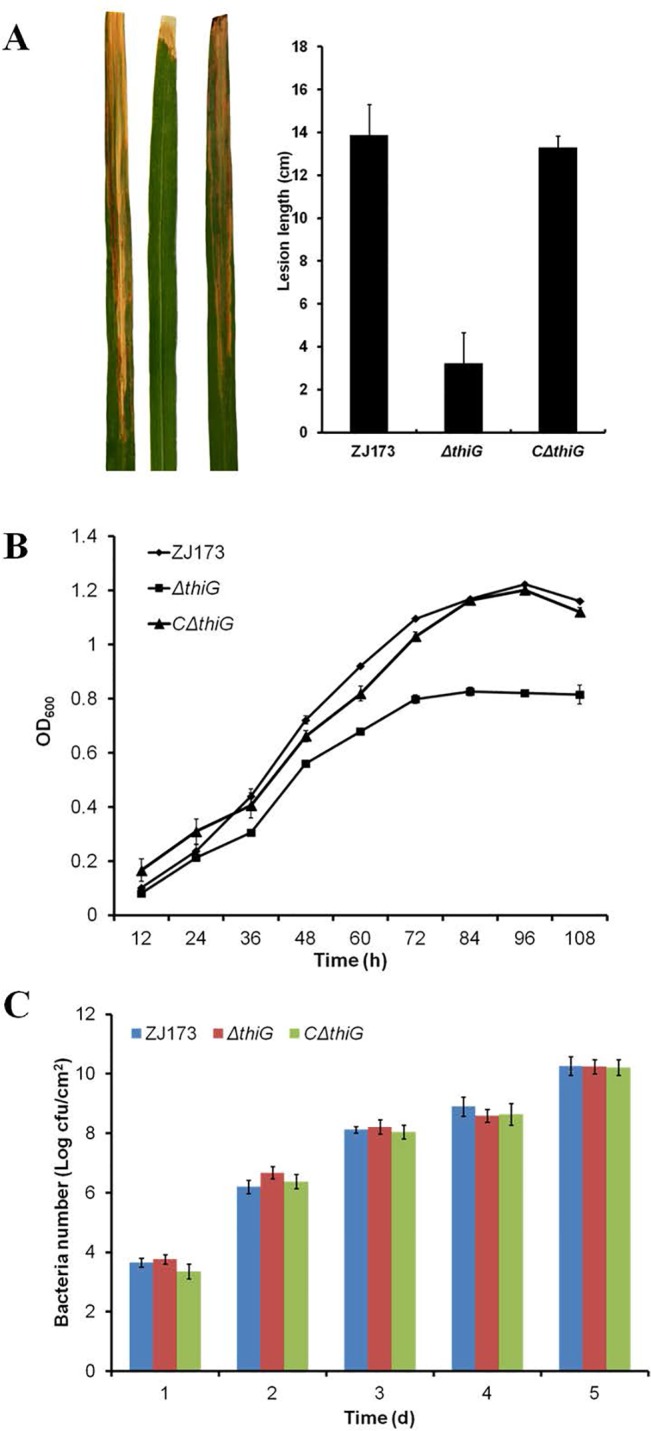
Effect of the *thiG* mutant on virulence and growth rates. (A) Representative leaves 14 days after inoculation by the leaf-clipping method and lesion lengths. (B) Growth rate of *Xoo* wild type strain ZJ173, the deletion mutant *ΔthiG* and the complemented strain *CΔthiG* in MMX nutrition limited medium. OD_600nm_, optical density at 600 nm. (C) Growth rate of *Xoo* wild type strain ZJ173, the deletion mutant *ΔthiG*, and the complemented strain *CΔthiG* in rice tissues. cfu, colony-forming unit. Vertical bars represent standard errors. Different letters above the data bars represents the significant value P<0.05.

To investigate whether *thiG* had an effect on the pathogenicity of *Xoo*, we examined the virulence of the *thiG* deletion mutant and its complemented strain *CΔthiG* on the susceptible rice cultivar IR24. Virulence was determined by lesion length at 14 days after inoculation. The data showed that the lesion length caused by *ΔthiG* was reduced compared to the wild-type strain ZJ173 and the complemented strain *CΔthiG* (Fig 2A). Although thiamine had important function in cellular metabolism and the deletion of *thiG* might be lethal in *Xoo*, the *thiG* deletion mutants only showed growth below that of the wild-type strain ZJ173 in the limited nutrition medium (MMX) without exogenous thiamine (Fig 2B). The growth rate of the deletion mutant was similar in the rich nutrition medium (NB), the MMX medium with thiamine supplementation (S1 Fig) and in infected rice tissue to that of the wild-type strain (Fig 2C). A similar phenomenon was also reported in the *thiG* mutant of *Fusarium oxysporum* and *Saccharomyces cerevisiae* [7, 8]. This implies that although the growth of the mutant was partially affected in the thiamine-free medium, normal growth patterns were restored when thiamine was present in extracellular environment, such as rice-leaf tissues [20]. In addition, we also found that several thiamine biosynthesis related genes were not required for the full virulence of *Xoo*. For example, the *thiL* deletion mutant exhibit a growth deficiency in the MMX medium but had similar virulence as the wild type strain in rice (S2 Fig). The protein encoded by *thiL* is required for thiamine phosphorylated to form TPP, which is the biologically active form of thiamine acting as the cofactor of key enzymes such as pyruvate dehydrogenase, a-ketoglutarate dehydrogenase, and others [21]. In *Salmonella typhimurium*, a *thi* operon (*thiBPQ*) is identified as ABC transport system to uptake exogenous thiamine and TPP [22], suggesting that the interruption of the thiamine biosynthesis pathway is not responsible for the reduction in virulence due to the potential thiamine transport system in *Xoo*. Taken together, the results indicate that the *thiG* gene is required for the full virulence of *Xoo* and that the virulence deficiency of the *thiG* mutant is not caused by an abnormal condition in the thiamine pathway but is the result of other virulence associated factors. Because the *thiG* mutant and wild-type strain grow more quickly in rich nutrition medium than in limited medium, we used the rich nutrition medium (NB) in the subsequent studies.

### Deletion of *thiG* in *Xoo* promoted the biofilm formation but suppressed the swarming motility of *Xoo* and bacterial migration in rice leaves

**Fig 3:**
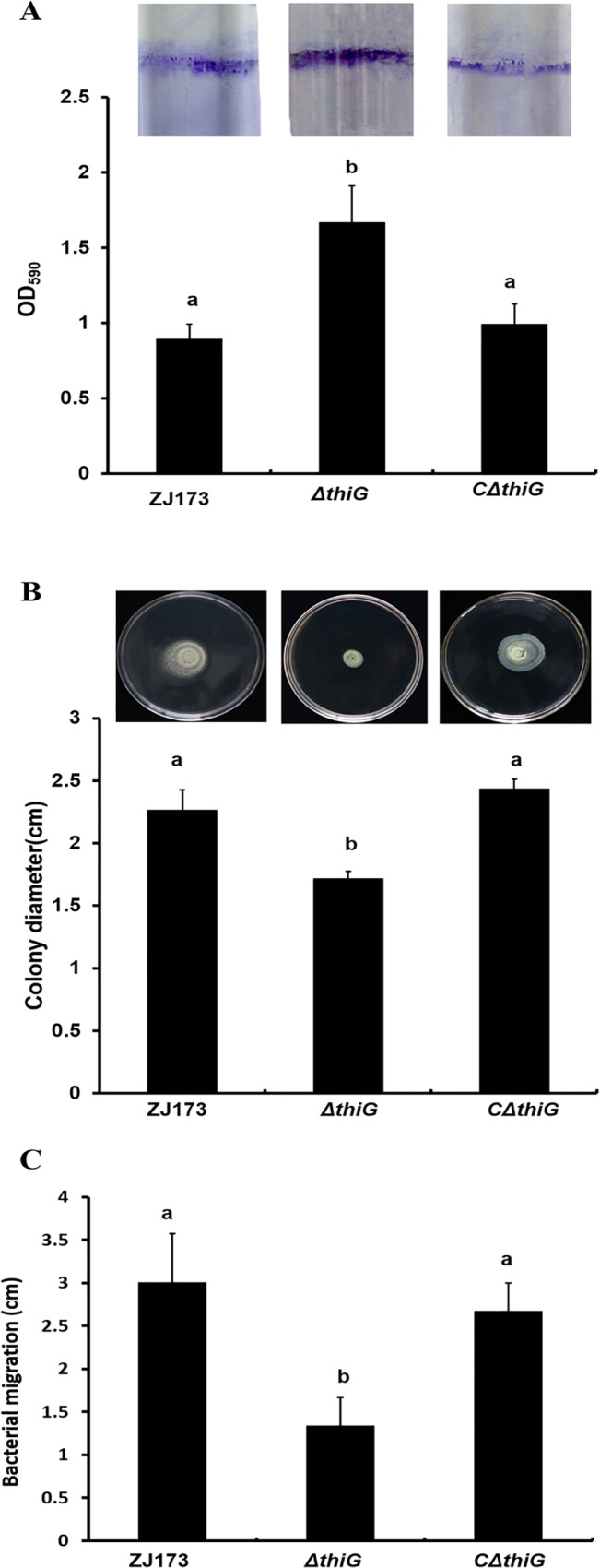
Effect of *thiG* mutant on biofilm formation and swimming motility. (A) Biofilm formation was increased in *ΔthiG*, and restored in the complemented strain *CΔthiG*. (B) The swimming motility was decrease in *ΔthiG*, and restored in the complemented strain *CΔthiG*. Vertical bars represent standard errors. Different letters above the data bars represents the significant value P<0.05.

Bacteria prefer to accumulating and forming cell-cell aggregation in natural environment rather than living as single cells in the medium. Biofilm, for example, which is associated with the chronic nature of subsequent infections and an increased capacity to tolerate environmental stresses [23]. The exopolysaccharide matrix of cells in biofilms is better at catching nutrients, concentrate enzymes and increasing metabolic activity compared with the cells under planktonic conditions. Meanwhile, these exopolysaccharide matrix is working as a physical barrier to protect the bacteria from environmental, biological, or chemical stresses [24]. Here, we evaluated the biofilm formation, swarming motility and bacterial migration to define the function of *thiG* in cell-cell aggregation in *Xoo*. Biofilm production of the wild-type strain ZJ173, the *thiG* mutant and its complemented strain were quantified at using crystal violet (CV) staining method. The results showed that the wild-type strain and its complemented strain generated half the amount of biofilm as the mutant *ΔthiG* (Fig 3A). The swarming motility of the mutant was determined by inoculating the bacteria onto a semi-solid NA medium plate. The mutant showed a decrease in swarming motility compared to the wild type strain, and restored in the complemented strain (Fig 3B). The bacterial migration assay was determined by inoculating the bacteria in the detached rice leaves and quantified the distance migrated within the leaves in 3d. The data showed that the distance migrated by *thiG* mutant is approximately half of the distance of the wild type strain ZJ173 and the complemented strain (Fig 3C). These results suggested that the cell-cell aggregation was affected in *thiG* deletion mutant. As mentioned above, the lesion length was reduced but the population of *ΔthiG* in the host was not affected, thereby we suggested that the loss of virulence is caused by the limit of infection progression.

Genes homologous to *thiG* in microorganisms were reported to be involved in increasing colony frequency and tolerance to both heat stress and oxidative stress [5, 7]. In addition, a genome-scale study of *Xac* biofilm formation showed 92 genes involved in biofilm formation including *thiG*, although they failed to demonstrate how biofilm formation was regulated by *thiG* [25]. These studies further support our conclusion that *thiG* is involved in cell-cell aggregation of *Xoo*. Since quorum sensing (QS) and extracellular substance are two key factors that contribute to the formation of cell aggregates, we further investigated these two aspects.

### The deletion of *thiG* negatively regulated the expression of other genes in thiamine biosynthesis pathway and the two-component system *rpfC*/*rpfG*

**Fig 4:**
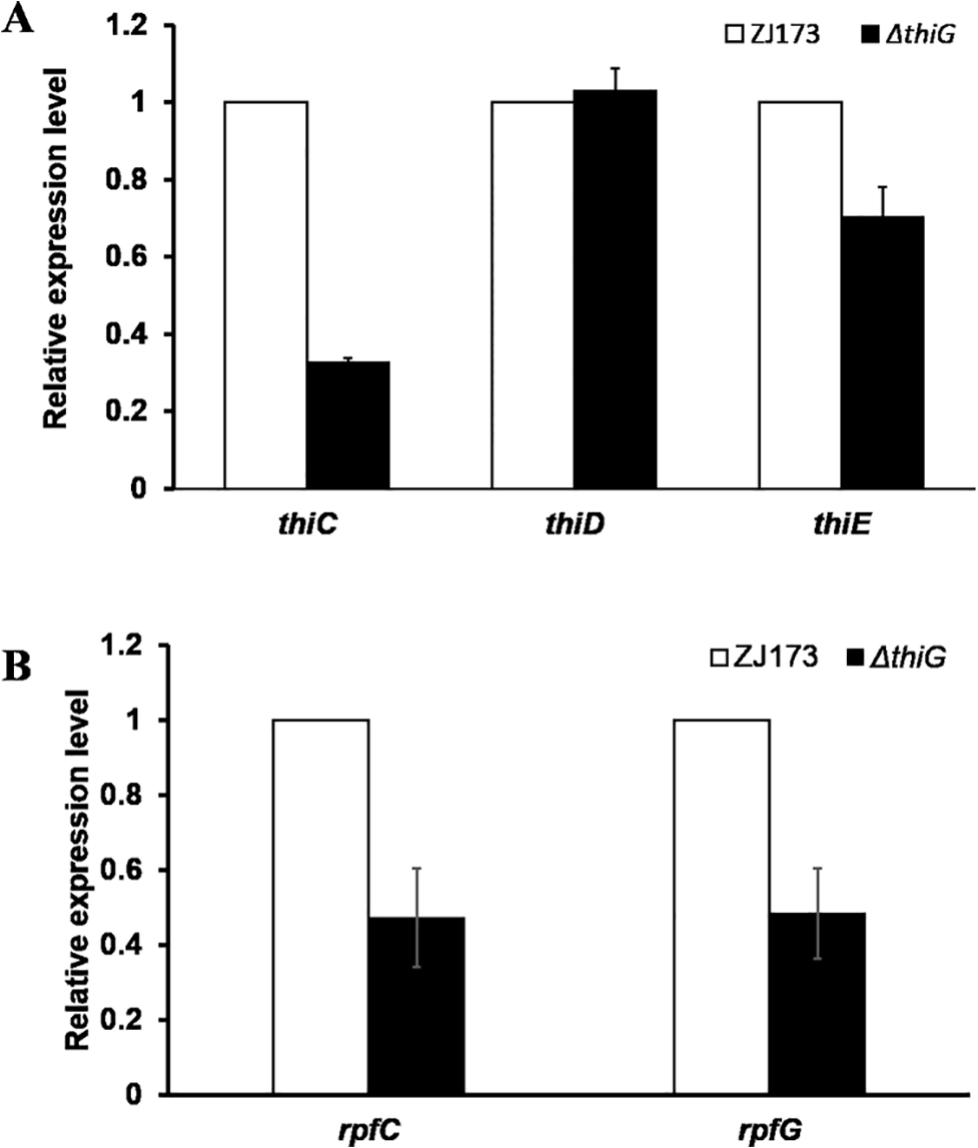
Regulation of the expression of other genes in thiamine biosynthesis and the two-conpoment regulatory genes, *rpfC*/*rpfG*, in the *thiG* deletion mutant. (A) The expression level of *thiC*, *thiD* and *thiE* in *ΔthiG* compare with the wild type strain ZJ173. (B) The expression of *rpfC* and *rpfG* in the mutant compared to the wild type strain ZJ173. Wild-type strain ZJ173 expression level was set as 1. *gyrB* was used as an internal control for data analyses. Vertical bars represent standard errors.

The cell aggregation and QS have closely relationship with each other. QS is a cell-cell communication mechanism that make bacteria recognize the population density by detecting the accumulation of specific signal factor which is secreted by bacteria [26]. In *Xcc*, diffusible signal factor (DSF) has been identified as signal molecule in QS system that regulated a lot of biological function, such as biofilm development and virulence [27]. The two-component system RpfC/RpfG has been implicated in DSF perception and signal transduction and can influence intracellular regulatory networks through c-di-GMP [28]. The *Xcc* mutants in the *rpfC*/*rpfG* system that growing in an aggregated fashion in culture failed to degrade c-di-GMP to two molecules of GMP leading to an increase in the cellular c-di-GMP level [29]. The alterations of c-di-GMP level may promote the binding to the cyclic-AMP receptor-like protein Clp, and then increase the production of ManA which is responsible for biofilm dispersing and repress xagABC expression which are required for exopolysaccharide synthesis [30]. Our data showed that the expression level of *rpfC* and *rpfG* were down regulated in the *thiG* deletion mutant compared to the wild type strain (Fig 4B), which means the RpfC/RpfG signaling system is involved in the formation of cells aggregates in the *thiG* deletion mutant.

Then we questioned why *thiG* is involved in the QS genes regulation. As mentioned above, AIR is a substrate of pyrimidine moiety biosynthesis in thiamine biosynthesis pathway, which is also an intermediate product in the purine biosynthesis pathway, and this pathway also generates c-di-GMP [31]. Considering the function of *thiG* in thiamine biosynthesis in *Xoo*, we hypothesized that other genes in the thiamine biosynthesis pathway may be affected in *thiG* deletion mutant and then indirectly modulate the intercellular c-di-GMP level. It has been proved that the expression of thiamine biosynthesis genes are induced in the absence of thiamine, and suppressed in the presence of thiamine in prokaryote and eukaryote [32]. In our study, all the *Xoo* strains are cultured in rich nutrition medium which means that the expression of thiamine biosynthesis genes should be repressed in *Xoo* due to the exogenous thiamine. However, our data showed that the expression of *thi* genes were affected and exhibit different regulation extent in the *thiG* mutant background. The expression of *thiC* and *thiE* is down-regulated compared with the wild type strain but *thiD* exhibit a similar regulation extent (Fig 4A). The study in *E*.*coli* showed that there are three thiamine biosynthesis operons in the genome (*thiCEFSGH*, *thiMD*, and *thiBPQ*), and each operons have one *thi* box sequence to regulate these genes translation [33]. It may be possible that *thiD* and *thiCE* genes is belong to two operons and controlled by respective *thi* box for translation leading to the different regulation tendency of these genes in the *thiG* mutant. Further analysis the linking of c-di-GMP mediated signaling network with *thiG*, we hypothesi3 that the expression of other *thi* genes is repressed in the *thiG* mutant background, which may cause the alteration of the intercellular c-di-GMP level and then the c-di-GMP mediated signaling network is modulated to cooperation with this alteration.

### The deletion of *thiG* had no effect of xanthan production but positively regulated some *gum* genes expression

**Fig 5:**
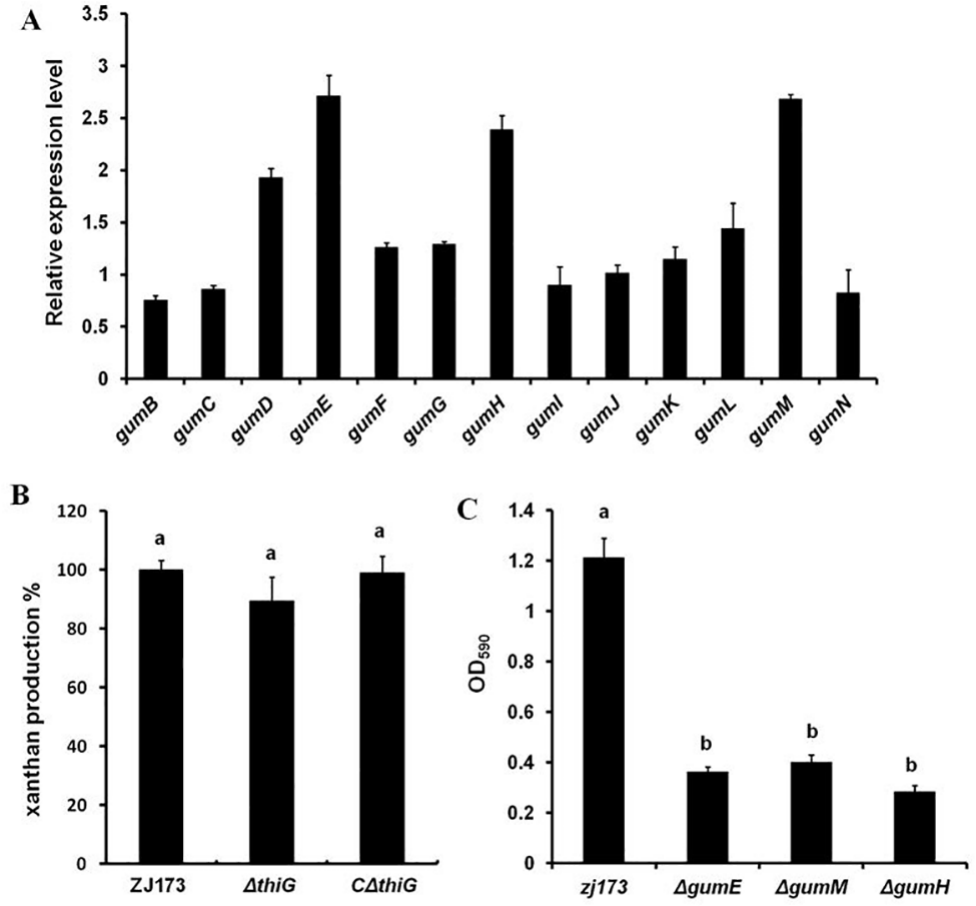
*thiG* is involved in regulating xanthan biosynthesis genes expression but had no effect on xanthan production. (A) The expression level of 13 *gum* genes in the *thiG* mutant compared to the wild type strain ZJ173.Wild-type strain ZJ173 expression level was set as 1. *gyrB* was used as an internal control for data analyses. (B) The xanthan production in the mutant compare with the wild-type strain ZJ173. Wild-type strain ZJ173 was set as equal to 100%. (C) Biofilm formation is decreased in *gumE*, *gumM* and *gumH* mutants compared to the wild-type strain ZJ173. Vertical bars represent standard errors. Different letters above the data bars represents the significant value P<0.05.

Flemming, H. C et al. (2007) pointed out if biofilms is a “city of microbes”, then EPS is just like the “house of the biofilm cells” [34]. In *Xcc*, the synthesis of the EPS xanthan plays important role in the integrity of cell aggregation. The xanthan possibly works on: interchain helix formation, chain–chain entanglements, interaction of the polysaccharide molecules with surface-attached components or interactions with other polysaccharide molecules [35]. EPS plays an important role in many cellular processes in microorganisms, like adhesion, aggregation of cells, pathogenicity, and so on [36]. In the Xanthononas genus, one of the most important EPS formations is xanthan, defined as pentasaccharide repeat units of glucose–glucose–mannose–glucuronate–mannose. The polysaccharides form as strands attached to the bacterial cell surface, which form a complex network surrounding the cell [37]. To investigate whether xanthan is involved in cell-cell aggregation in the *thiG* mutant, we examined the xanthan production in the wild type strain ZJ173, the mutant *ΔthiG* and its complemented strain *CΔthiG*. Unexpectedly, the production of xanthan in *ΔthiG* showed no significant difference compared to ZJ173 and *CΔthiG* (Fig 5B). Although EPS may promote adherence to a wide variety of solid substrate, its structures also play a crucial role in the cells aggregation. In oral biofilms, EPS structures provide a binding location for other oral bacteria and then permit accretion of cells. The poorly water-soluble α-D-glucose molecules are secreted first and attach to the oral surfaces. Oral streptococci can then bind subsequently, therefore making glucose act as intra-molecular bridge [38]. Xanthan biosynthesis and export is regulated by the *gum* gene cluster, which is composed of 13 genes (*gumB* to *gumN*) in *Xoo* [39]. The transcriptional expression of *gum* genes was identified and analyzed in the *thiG* mutant by RT-PCR. The data showed that the expression of most of the *gum* genes is not significantly altered in the *thiG* mutant, however, the *gumD*, *gumE*, *gumH* and *gumM* genes are up-regulated more than two fold in the *thiG* deletion mutant compared to the wild type strain (Fig 5A). Pervious literature reports that GumD and GumM are the initial and second glycosyltransferase, which are responsible for the transfer of a glucose moiety to form a 1, 4-linked glucose-disaccharide with the lipid carrier, and that GumH transfers GDP-mannose to the disaccharide with 1, 3-glycosidic link, and that GumE is possible for the polymerase function [37]. Zhang *et al*. showed that the deletion of *gumD* down regulates the biofilm formation in *Xoc* [40]. Our study found that the biofilm formation in *gumE*, *gumH* and *gumM* deletion mutants are also repressed (Fig 5C), suggesting that *gumE*, *gumH* and *gumM* may positively regulate biofilm formation. The transcriptional regulation of these *gum* genes indicated that the structure of xanthan could be influenced in the *thiG* mutant even though the production was not changed, which lead to the formation of cell-cell aggregates. Whether the *thiG* gene regulates these genes directly or indirectly needs to be further investigated.

## Conclusion

The findings in this study demonstrated that the *thiG* gene, which is involved in the thiazole moiety production in thiamine biosynthesis pathway, is required for the full virulence of *Xoo* on rice. The knockout of *thiG* in *Xoo* caused a decrease in growth rate in limited nutrition medium, but not in the host. Meanwhile, the formation of cell-cell aggregates like biofilm formation, swarming motility and bacterial migration in rice were influenced in the *thiG* mutant. These results suggest that the loss of virulence maybe caused by a reduction in the infection progression in rice. Moreover, the deletion of *thiG* suppressed the expression level of the *rpfC* and *rpfG*, which might indirectly induce abnormal regulation of thiamine biosynthesis in the *thiG* mutant. Xanthan production was not changed in the mutant but the expression of some *gum* genes were up-regulated, but how the absence of *thiG* affects the regulation of *gum* gene expression needs further study. These results provide new insight about role of the *thiG* gene in *Xoo* virulence and serve as an example for future research to investigate alternative functions of genes in vitamin biosynthesis pathways.

## Supporting Information

S1 Fig The growth ability of the *thiG* mutant in the rich nutrition medium and the limited nutrition medium with thiamine supplementation. (A) Growth rate of *Xoo* wild type strain ZJ173 and the deletion mutant *ΔthiG* in NB nutrition rich medium. (B) The growth ability of *Xoo* wild type strain ZJ173 and the deletion mutant *ΔthiG* in nutrition limited medium MMX with 0, 0.5, 5, 10, 20 μg/ml thiamine. The growth deficiency of *ΔthiG* is restored in MMX medium by thiamine supplementation. OD_600nm_, optical density at 600 nm. Vertical bars represent standard errors.
(DOCX)

S2 Fig Effect of the *thiL* mutant on virulence and growth rates. (A) Representative leaves 14 days after inoculation by the leaf-clipping method and lesion lengths. (B) Growth rate of *Xoo* wild type strain ZJ173, the deletion mutant *ΔthiL* in MMX nutrition limited medium. The *thiL* deletion mutant exhibit a growth deficiency in the MMX medium but had similar virulence as the wild type strain in rice. OD_600nm_, optical density at 600 nm. Vertical bars represent standard errors. Different letters above the data bars represents the significant value P<0.05.
(DOCX)

S1 Table Primers used in this study. (DOC)
