# Supplementary material for: The thiG Gene Is Required for Full Virulence of Xanthomonas oryzae pv. oryzae by Preventing Cell Aggregation
Source: PLoS One. 2015 Jul 29;10(7):e0134237. doi: 10.1371/journal.pone.0134237 (PMC4519133; doi:10.1371/journal.pone.0134237)
Supplement: S1 Fig — (A) Growth rate of Xoo wild type strain ZJ173 and the deletion mutant ΔthiG in NB nutrition rich medium. (B) The growth ability of Xoo wild type strain ZJ173 and the deletion mutant ΔthiG in nutrition limited medium MMX with 0, 0.5, 5, 10, 20 μg/ml thiamine. The growth deficiency of ΔthiG is restored in MMX medium by thiamine supplementation. OD600nm, optical density at 600 nm. Vertical bars represent standard errors. (DOCX) [file pone.0134237.s001.docx]

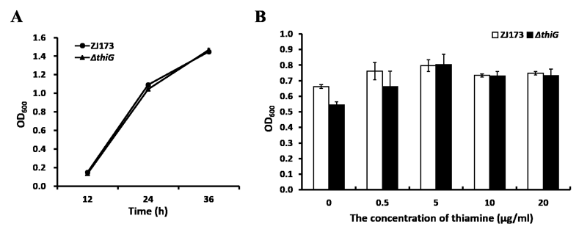

**S1 Fig. The growth ability of the *thiG* mutant in the rich nutrition medium and the limited nutrition medium with thiamine supplementation.** (A) Growth rate of *Xoo* wild type strain ZJ173 and the deletion mutant *ΔthiG* in NB nutrition rich medium. (B) The growth ability of *Xoo* wild type strain ZJ173 and the deletion mutant *ΔthiG* in nutrition limited medium MMX with 0, 0.5, 5, 10, 20 µg/ml thiamine. The growth deficiency of *ΔthiG* is restored in MMX medium by thiamine supplementation. OD_600nm_, optical density at 600 nm. Vertical bars represent standard errors.
