# Supplementary material for: The thiG Gene Is Required for Full Virulence of Xanthomonas oryzae pv. oryzae by Preventing Cell Aggregation
Source: PLoS One. 2015 Jul 29;10(7):e0134237. doi: 10.1371/journal.pone.0134237 (PMC4519133; doi:10.1371/journal.pone.0134237)
Supplement: S2 Fig — (A) Representative leaves 14 days after inoculation by the leaf-clipping method and lesion lengths. (B) Growth rate of Xoo wild type strain ZJ173, the deletion mutant ΔthiL in MMX nutrition limited medium. The thiL deletion mutant exhibit a growth deficiency in the MMX medium but had similar virulence as the wild type strain in rice. OD600nm, optical density at 600 nm. Vertical bars represent standard errors. Different letters above the data bars represents the significant value P<0.05. (DOCX) [file pone.0134237.s002.docx]

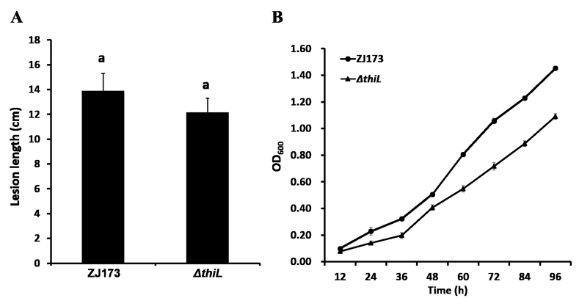


S2 Fig. **Effect of the *thiL* mutant on** **virulence and growth rates.** (A) Representative leaves 14 days after inoculation by the leaf-clipping method and lesion lengths. (B) Growth rate of *Xoo* wild type strain ZJ173, the deletion mutant *ΔthiL* in MMX nutrition limited medium. The *thiL* deletion mutant exhibit a growth deficiency in the MMX medium but had similar virulence as the wild type strain in rice. OD_600nm_, optical density at 600 nm. Vertical bars represent standard errors. Different letters above the data bars represents the significant value P<0.05.
