# Supplementary material for: The thiG Gene Is Required for Full Virulence of Xanthomonas oryzae pv. oryzae by Preventing Cell Aggregation
Source: PLoS One. 2015 Jul 29;10(7):e0134237. doi: 10.1371/journal.pone.0134237 (PMC4519133; doi:10.1371/journal.pone.0134237)
Supplement: S1 Table — (DOC) [file pone.0134237.s003.doc]

Table S1. Primers used in this study

| Purpose | Primes | Sequence (5’→3’; restriction sites underlined) | Description |
| --- | --- | --- | --- |
| Mutagenesis and Complementation | *thiG*up-F  *thiG*up-R | CGGGATCCGGAGTGCCTGCTTTGA GGGGTACCGGGTACGTCCTGTCGA | A 664 bp fragment upstream to *thiG* |
|  | *thiG*down-F  *thiG*down-R | GGGGTACCAGAGGGGCTTCTTTCAG  GCGTCGACGCGGCTTGGGTCTT | A 370 bp fragment downstream to *thiG* |
|  | *thiG-*cF  *thiG-*cR | GGGGTACCTTGTTGTTTCGTTGGAGGGA  CGGGATCCTCATCCAATCACGCCATCCA | To amplify *thiG* gene and its promoter from *Xoo* strain ZJ173 |
| Southern Blot | *thiG*-southernF  *thiG*-southernR | GCGGATCGGGCAAAGTGTT  AGGGGGCGGTCTCAAGAGT | A 599 bp fragment of *thiG* was used as a probe for southern blot |
| Real-time quantitative RT-PCR | *thiC*-F  *thiC*-R | CTCTGCTATGTCACTCCGAAAG  GCGATCTTGTAGGCCATGAT | A 83 bp fragment of *thiC* |
|  | *thiD*-F:  *thiD*-R: | GTGACCCAGGATGTGTTCAT  AGGCATTGAGCAACGACA | A 118 bp fragment of *thiD* |
|  | *thiE*-F  *thiE*-R | GGTTGCAATACCGCAACAAG  ATGATCAATGGCACACCGT | A 100 bp fragment of *thiE* |
|  | *gumB*-F  *gumB*-R | ATCGTGTTCCGCATGGTC  ATATCGCCGCCGTAAATCTC | A 104 bp fragment of *gumB* |
|  | *gumC*-F  *gumC*-R | GAAGCAAAGCTCGACAAGGA  GACGCGAATCCACCGATTTA | A 106 bp fragment of *gumC* |
|  | *gumD*-F  *gumD*-R | GCGTGCGATTCGTATGTTTC  GCGAGGAAGCTGTGTAAGT | A 106 bp fragment of *gumD* |
|  | *gumD*-F  *gumD*-R | GCGTGCGATTCGTATGTTTC  GCGAGGAAGCTGTGTAAGT | A 102 bp fragment of *gumD* |
|  | *gumE*-F  *gumE*-R | TGCTGTTGATGCCCAGTAAG  CTTGATCGAGAAGTAGCCGTTG | A 101 bp fragment of *gumE* |
|  | *gumF*-F  *gumF*-R | TAGCTTGCTGCTGCCATAC  CAGCCTTCTCACCGACATTAC | A 83 bp fragment of *gumF* |
|  | *gumG*-F  *gumG*-R | GCCAATGGTTCACGTCTGTA  GCACGCAAGGCAATGTAAG | A 89 bp fragment of *gumG* |
|  | *gumH*-F  *gumH*-R | GGTACTCGATGCGATTCGTT  GGCTTGGTCAATGCCAGATA | A 84 bp fragment of *gumH* |
|  | *gumI*-F  *gumI*-R | GCTGCACTTCGGCCTGATTC  GCACCGGCTCTTCCACATAG | A 121 bp fragment of *gumI* |
|  | *gumJ*-F  *gumJ*-R | CGCGATAACCTGTTCTGGAT  GCCGGCTCTTTGTAGAAGT | A 101 bp fragment of *gumJ* |
|  | *gumK*-F  *gumK*-R | CGGTGTACTTGGCTGATAGTT  AGCCGAAACGCGATTGATA | A 108 bp fragment of *gumK* |
|  | *gumL*-F  *gumL*-R | GTAACAGCGTGGAAGAGTGT  CACCAAAGGTCGTAAGGGAAT | A 103 bp fragment of *gumL* |
|  | *gumM*-F  *gumM*-R | TGTTGAGACGACGGGAATTG  CTGCCAACGCATGGAATAGA | A 125 bp fragment of *gumM* |
|  | *gumN*-F  *gumN*-R | GACAGGCGCTCAAGGAAT  TCCACCATCGTAGGCAAATC | A 100 bp fragment of *gumN* |
|  | *rpfC*-F  *rpfC*-R | CCGTTTCCTGGCCAATATGA  CAGGCATTCCTTCTGCTCTAC | A 112 bp fragment of *rpfC* |
|  | *rpfG*-F  *rpfG*-R | GACGAGATGAGCGTGATGAA  GATAACCGGTGCCGTCATAA | A 133 bp fragment of *rpfG* |
|  | *gyrB*-F  *gyrB*-R | CCTGTTGCTGACCTTCTTCTAC  CTTCAGATACAGCTCGCTCTTG | A 118 bp fragment of *gyrB* |

^a^Restricted digestion enzyme site was underlined.
